# Supplementary material for: Exploring the Toxic Effects of ZEA on IPEC-J2 Cells from the Inflammatory Response and Apoptosis
Source: Animals (Basel). 2023 Aug 28;13(17):2731. doi: 10.3390/ani13172731 (PMC10487149; doi:10.3390/ani13172731)
Supplement: Supplementary file 1 [file animals-13-02731-s001.zip › animals-2575332-supplementary.pdf]

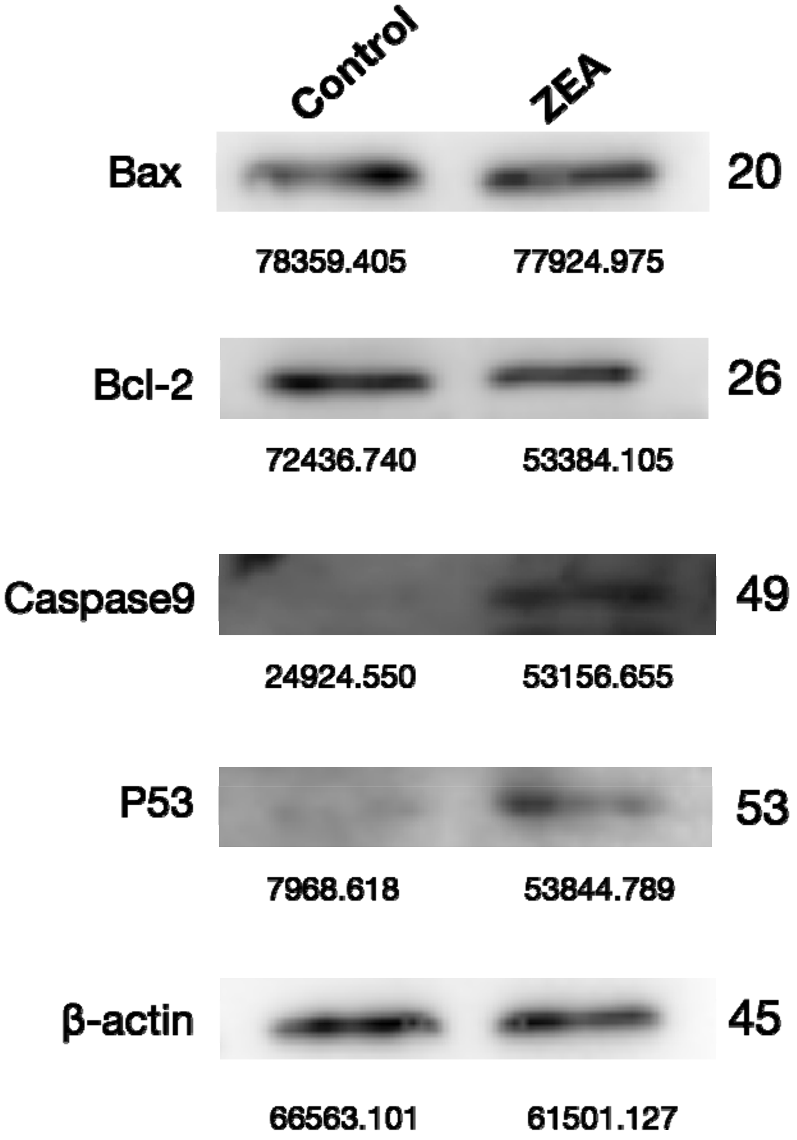

Figure S1. Inflammation-related gene protein expression results are plotted.（The value marked between each band is the grey scale value of that band, and the molecular weight size of the protein is marked on the right side of the band.）

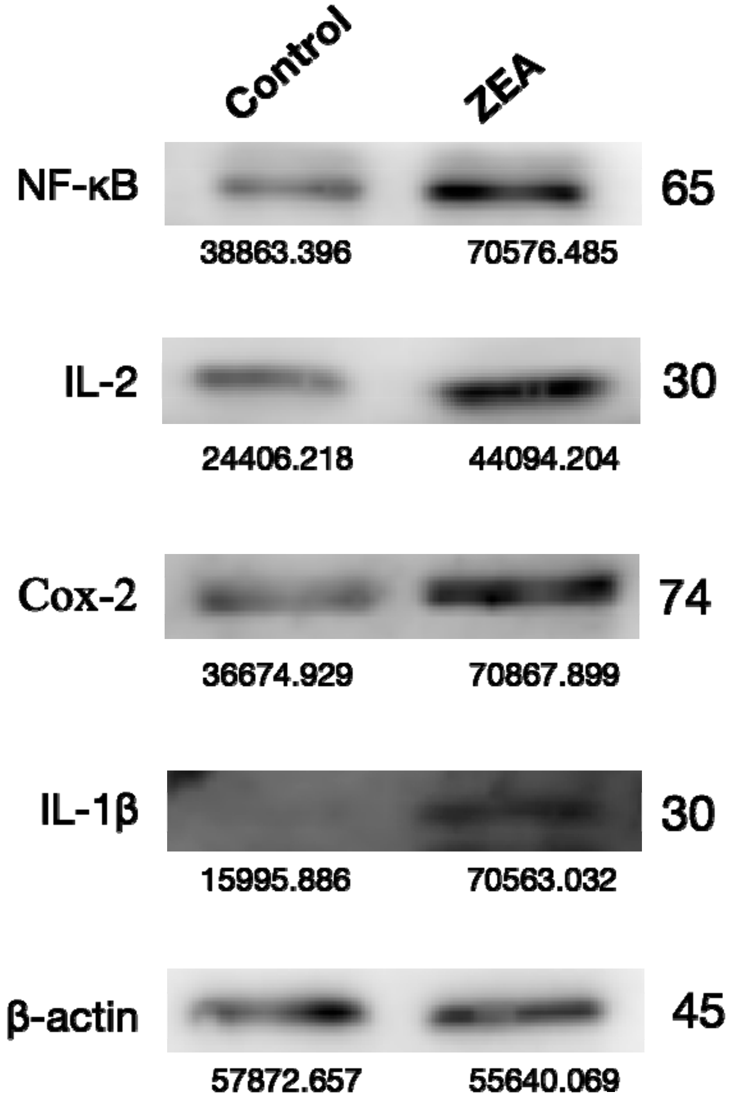

Figure S2. Protein expression results of apoptosis-related genes map。（The value marked between each band is the grey scale value of that band, and the molecular weight size of the protein is marked on the right side of the band.）
